# Hepatic triglyceride content does not affect circulating CETP: lessons from a liraglutide intervention trial and a population-based cohort

**DOI:** 10.1038/s41598-019-45593-2

**Published:** 2019-07-10

**Authors:** Huub J. van Eyk, Lisanne L. Blauw, Maurice B. Bizino, Yanan Wang, Ko Willems van Dijk, Renée de Mutsert, Johannes W. A. Smit, Hildo J. Lamb, Ingrid M. Jazet, Patrick C. N. Rensen

**Affiliations:** 1 Department Medicine, Div. Endocrinology, Leiden University Medical Center (LUMC), Leiden, The Netherlands; 2 Einthoven Laboratory for Experimental Vascular Medicine, LUMC, Leiden, The Netherlands; 3 Department Epidemiology, LUMC, Leiden, The Netherlands; 4 Department Radiology, LUMC, Leiden, The Netherlands; 5 Department Human Genetics, LUMC, Leiden, The Netherlands; 6 Department Medicine, Radboud University Medical Center, Nijmegen, The Netherlands

**Keywords:** Endocrine system and metabolic diseases, Endocrine system and metabolic diseases

## Abstract

Cholesteryl ester transfer protein (CETP) is mainly expressed by Kupffer cells in the liver. A reduction of hepatic triglyceride content (HTGC) by pioglitazone or caloric restriction is accompanied by a decrease in circulating CETP. Since GLP-1 analogues also reduce HTGC, we assessed whether liraglutide decreases CETP. Furthermore, we investigated the association between HTGC and CETP in a population-based cohort. In a placebo-controlled trial, 50 patients with type 2 diabetes were randomly assigned to treatment with liraglutide or placebo added to standard care. In this trial and in 1,611 participants of the Netherlands Epidemiology of Obesity (NEO) study, we measured HTGC and circulating CETP by proton magnetic resonance spectroscopy and ELISA, respectively. The HTGC was decreased in the liraglutide group (−6.3%; 95%CI of difference [−9.5, −3.0]) but also in the placebo group (−4.0%; 95%CI[−6.0, −2.0]), without between-group differences. CETP was not decreased by liraglutide (−0.05 µg/mL; 95%CI[−0.13, 0.04]) or placebo (−0.04 µg/mL; 95%CI[−0.12, 0.04]). No association was present between HTGC and CETP at baseline (β: 0.002 µg/mL per %TG, 95%CI[−0.005, 0.009]) and between the changes after treatment with liraglutide (β: 0.003 µg/mL per %TG, 95%CI[−0.010, 0.017]) or placebo (β: 0.006 µg/mL per %TG, 95%CI[−0.012,0.024]). Also, in the cohort n o association between HTGC and CETP was present (β: −0.001 µg/mL per SD TG, 95%CI[−0.005, 0.003]). A reduction of HTGC after treatment with liraglutide or placebo does not decrease circulating CETP. Also, no association between HTGC and CETP was present in a large cohort. These findings indicate that circulating CETP is not determined by HTGC.

Clinical Trial Registration: Clinicaltrials.gov (NCT01761318).

## Introduction

Cholesteryl ester transfer protein (CETP) facilitates the transfer of cholesteryl esters from HDL towards triglyceride-rich lipoproteins, mainly VLDL, coupled to a net flux of triglycerides from VLDL to HDL^1^. CETP thereby causes a proatherogenic lipoprotein profile, with increased atherogenicity of VLDL particles^2^. Recently, it has been shown that circulating levels of CETP are mainly determined by Kupffer cells, which are the resident macrophages of the liver^3,4^, and that adipose tissue does not relevantly contribute to serum CETP concentration^5^.

Although Kupffer cells in the liver have been identified as the main source of circulating CETP, the regulation of hepatic CETP production is incompletely understood. Previous human studies showed that treatment with the peroxisome proliferator-activated receptor (PPAR)-γ agonist pioglitazone^6^ and prolonged caloric restriction^7^ both reduced hepatic triglyceride content, which was accompanied by a decrease in plasma CETP concentration. Interestingly, it was recently shown that treatment with liraglutide, a human glucagon-like peptide 1 (GLP-1) analogue, also reduces hepatic steatosis and can even lead to histological resolution of non-alcoholic steatohepatitis (NASH)^8^. GLP-1 analogues are prescribed to patients with type 2 diabetes to achieve glycemic control, whereby they also induce weight loss^9,10^. However, the exact mechanism of action by which GLP-1 analogues ameliorate hepatic steatosis and NASH is still unclear. In rodents, GLP-1 analogues were shown to improve diet-induced hepatic steatosis and reduce hepatic macrophage recruitment^11,12^. Notably, the GLP-1 analogue exendin-4 not only decreased hepatic triglycerides but also hepatic *CETP* gene expression and circulating CETP concentration in human CETP expressing transgenic mice^12^. Since we previously showed that hepatic *CETP* expression and plasma CETP concentration were strongly related with the hepatic Kupffer cell content^3^, the reduction in CETP observed with exendin-4 treatment may be explained by a reduction in Kupffer cells. However, in humans, the effects of GLP-1 analogues on CETP production are still unknown.

The aim of the present study was to assess whether a liraglutide-induced reduction in hepatic triglyceride content would be accompanied by a reduction in circulating CETP concentration in patients with type 2 diabetes. In addition, we also investigated the association between hepatic triglyceride content and circulating CETP concentration in a population-based cohort of 1,611 participants.

## Materials and Methods

### Randomised controlled trial

#### Study overview and study population

This study used data from the MAGNA VICTORIA (MAGNetic resonance Assessment of VICTOza efficacy in the Regression of cardiovascular dysfunction In type 2 diAbetes mellitus) study, a prospective, randomised, double-blind, clinical trial. The primary outcome measure of this study was the effect of liraglutide on cardiac function^13^. In the current manuscript, we report on secondary and other endpoints. Overweight and obese (BMI ≥ 25 kg/m^2^) patients with type 2 diabetes were recruited from November 2013 until March 2016 via advertisements and from the outpatient clinics of the Leiden University Medical Center (LUMC, Leiden, the Netherlands), general practitioners, and local hospitals. We included patients aged 18–69 years, treated with metformin, and with a glycated haemoglobin (HbA1c) ≥ 7.0 and ≤10.0% (53–86 mmol/mol). Concomitant treatment with sulfonylurea derivatives and insulin was optional, although the dosage of all glucose-lowering medication needed to be stable for at least 3 months prior to participation. Exclusion criteria were use of other glucose-lowering therapy than mentioned above, presence of renal, hepatic or cardiovascular disease, and contra-indications for magnetic resonance imaging (MRI). The trial was approved by the ethics committee of the LUMC and performed in accordance with the principles of the revised Declaration of Helsinki. Written informed consent was obtained from all subjects before participation. The trial was conducted at the LUMC, and was registered at clinicaltrials.gov (NCT01761318, date of registration 04/01/2013).

#### Study design and data collection

At baseline, participants were randomised to receive treatment with liraglutide (Victoza®, Novo Nordisk A/S, Bagsvaerd, Denmark) or placebo by block randomisation with block size of 4 and stratification 1:1 for sex and insulin use. During the study, all participants, study investigators and outcome assessors were blinded to treatment allocation. Participants visited the study center at baseline and after 26 weeks of treatment, after ≥6 h of fasting, for medical history assessment, standard physical examination, collection of venous blood samples and MRI. After 4 and 12 weeks of treatment additional venous blood samples were collected. The starting dose of the study medication was 0.6 mg per day, which was titrated in two weeks to a maximum dose of 1.8 mg per day, if tolerated. In addition to study medication, participants received treatment according to current clinical guidelines to achieve optimal glycemic control and regulation of blood pressure and cholesterol levels. Body composition was assessed using bioelectrical impedance analysis (BIA; Bodystat 1500, Bodystat Ltd., Douglas, UK). All blood samples were centrifuged and stored at −80 °C until analysis. Plasma cholesterol and triglyceride concentrations were measured on a Modular P800 analyser (Roche Diagnostics, Mannheim, Germany). LDL-cholesterol was calculated according to Friedewald’s formula^14^. Due to changes in laboratory procedures during the study, in a subset of participants HbA1c was assessed with boronate affinity high-performance liquid chromatography (Primus Ultra, Siemens Healthcare Diagnostics, Breda, the Netherlands), while in the other patients HbA1c was assessed with ion-exchange high-performance liquid chromatography (HPLC) (Tosoh G8, Sysmex Nederland B.V., Etten-Leur, the Netherlands). Plasma CETP concentrations were determined by ELISA slightly modified from Niemeijer-Kanters *et al*.^15^. In short, plates were coated with a combination of monoclonal antibodies TP1 (5 µg/ml) and TP2 (2.5 µg/mL; both from Ottawa Heart Institute Research Corporation, Ottawa, Canada) during an overnight incubation at 4 °C. The next day plates were blocked with 1% BSA (Sigma Aldrich, Zwijndrecht, the Netherlands) for 2 hours at room temperature. EDTA-plasma samples were 80-fold diluted in Assay buffer containing 1% BSA and 0.1% Triton-X100 (Biorad, Veenendaal, the Netherlands) and incubated for 2 hours at 37 °C. Autocal (Instruchemie, Delfzijl, the Netherlands) was used as a standard. Subsequently, plates were incubated with the secondary antibody TP20 labeled with digoxigenin (0.33 µg/mL; Ottawa Heart Institute Research Corporation, Ottawa, Canada) for 2 hours at 37 °C, followed by 1 hour of incubation with anti-digoxigenin-POD, Fab fragments coupled to peroxidase (0.0375 U/mL; Roche Molecular Biochemicals, Mannheim, Germany) at room temperature. Finally, plates were incubated with TMB for 15 minutes and, after termination of the reaction with H_2_O_2_, absorbance was read at 450 nm. The interassay variance was <10% and the intraassay variance was <5%.

#### Proton magnetic resonance spectroscopy

Hepatic triglyceride content was assessed with proton magnetic resonance spectroscopy (^1^H-MRS) on a 3 Tesla Ingenia whole-body MR system (Philips Medical Systems, Best, the Netherlands). Subjects were scanned in supine position after at least 6 hours of fasting. The body coil was used for transmission and reception was achieved with a 16-element anterior, and a 12-element posterior array. The 20 × 20 × 20 mm^3^ voxel of interest (VOI) was placed in the liver while carefully avoiding contamination of bile ducts and blood vessels. VOI localization was achieved using a PRESS (point resolved spectroscopy) sequence, with echo time of 35 ms and repetition time of 9 s for the unsuppressed spectra and 3.5 s for the water-suppressed spectra. First order pencil beam B_0_ shimming, with nine projections, was performed in the spectroscopic VOI. Four signal averages (NSA) were acquired without water suppression, and 32 NSA with water suppression using the MOIST (Multiply Optimized Insensitive Suppression Train) sequence. Spectra were acquired during free-breathing at end-expiration with pencil beam navigator-based respiratory triggering technique. The navigator voxel was placed at the lung-liver interface. Further movement artefacts were minimalized by applying motion tracking that corrected the voxel location according to the navigator position. The excitation bandwidth was 1500 Hz and 1024 samples were acquired resulting in spectral resolution of 1.46 Hz/sample. The raw spectral data were processed by an in-house developed program that performed channel weighting, phase correction and frequency drift correction. After these steps, signal averages that were >2.5 times the standard deviation were excluded. Lastly the remaining averages were summed after which the spectra were fitted in the time-domain using the Java-based MR User Interface ((jMRUI version 5.0; Katholieke Universiteit Leuven, Leuven, Belgium). Before fitting the spectrum residual water signal for the water-suppressed spectra was removed using a Hankel-Lanczos singular value decomposition (HLSVD) filter. The advanced method for accurate, robust and efficient spectral fitting (AMARES) algorithm was used to fit the resonances to a Gaussian line shape. Hepatic triglyceride content was calculated by dividing sum of triglyceride methyl (CH_3_) and triglyceride methylene (CH_2_) by water + CH_3_ + CH_3_ * 100%. All spectra were blinded before analysis.

#### Statistical analyses

Data are shown as means ± SD, or as median (interquartile range) when not normally distributed. Within-group changes were assessed using paired *t-*tests. We performed an analysis of covariance (ANCOVA) to assess between-group differences. Linear regression analyses were performed to determine associations between hepatic triglyceride content and plasma CETP concentration, and between the change (Δ) in hepatic triglyceride content and the change in plasma CETP level. β and corresponding 95% CI were reported. A *P-*value < 0.05 was considered statistically significant. Statistical analyses were performed using SPSS version 23.0 for Windows (IBM Corporation, Chicago, IL).

### Population-based study

#### Study overview and study population

The study population was part of the Netherlands Epidemiology of Obesity (NEO) study, which is a population-based prospective cohort study of 6,671 men and women between 45 and 65 years, with an oversampling of persons with a BMI of 27 kg/m^2^ or higher. Participants were recruited from September 2008 until September 2012, and visited the NEO study center after an overnight fast of at least 10 h for extensive baseline measurements, including venous blood sampling and anthropometry. In a random subgroup of 2,082 participants hepatic triglyceride content was available. The present study is a cross-sectional analysis of the baseline measurements of the NEO study. We excluded participants with missing data on serum CETP concentration (n = 16), or high alcohol intake according to criteria from the World Gastroenterology Organisation^16^, i.e. >30 g/day for men and >20 g/day for women. Therefore, the present study population comprised 1,611 NEO study participants who underwent ^1^H-MRS of the liver. The NEO study was approved by the medical ethics committee of the LUMC and all participants gave written informed consent. More detailed information about the study design and data collection has been described previously^17^.

#### Data collection

Hepatic triglyceride content was quantified by ^1^H-MRS on a 1.5 Tesla MR system (Philips Medical Systems, Best, the Netherlands). CETP concentrations were measured with ELISA kits according to the manufacturer’s instructions (DAIICHI CETP ELISA, Daiichi, Tokyo, Japan), in serum that had undergone one previous freeze-thaw cycle. Fasting concentrations of ALT and AST were measured with a Cobas Integra 800 analyzer (Roche Diagnostics, Mannheim, Germany). More detailed information on covariates can be found in the supplementary information.

#### Statistical analyses

Linear regression analyses were performed to examine the association between hepatic triglyceride content and serum CETP concentration. All results from the NEO study were based on analyses weighted towards a reference BMI distribution of the general population, and therefore apply to a population-based study without oversampling of individuals with overweight or obesity (see supplementary information). As hepatic triglyceride content was not normally distributed, this variable was transformed to the natural logarithm. For the purpose of interpretation, beta coefficients from linear regression analyses were multiplied by ln(1.1), and the difference in serum CETP concentration with corresponding 95%CI was expressed per 10% relative increase in hepatic triglyceride content. The crude model (Model 1) was adjusted for age and sex (Model 2), and subsequently ethnicity, smoking status, alcohol intake and physical activity were added as confounding variables (Model 3). Analyses were performed using STATA Statistical Software (Statacorp, College Station, Texas, USA), version 12.0.

## Results

### Randomised controlled trial

#### Population characteristics

Participants were included between December 2013 and September 2015 with the last participant visiting in March 2016. Fifty participants were included, of whom 24 were randomised to receive liraglutide and 26 to receive placebo. One participant of the liraglutide group was withdrawn from the study before starting treatment due to claustrophobia and was not included in the analyses. Another participant of the liraglutide group did not finish the study due to misdiagnosis of type 2 diabetes and one patient of the placebo group was lost to follow-up due to imprisonment, but the baseline measurements of both participants were used for analyses. Three serious adverse events occurred that were not related to study drug use. As shown in Table 1, baseline characteristics of the participants in both treatment groups were comparable. Individuals were 59.9 ± 6.2 years old in the liraglutide group, *vs* 59.2 ± 6.8 years in the placebo group, with a body weight of 98.4 ± 13.8 *vs* 94.5 ± 13.1 kg and BMI of 32.6 ± 4.4 *vs* 31.6 ± 3.4 kg/m^2^, respectively. Of all 49 participants included in the study, 40 participants used lipid lowering drugs at the start of the study.

**Table 1:** Baseline characteristics of participants from the MAGNA VICTORIA study.

Characteristic	Liraglutide (n = 23)	Placebo (n = 26)
**Demographics**		
Age (years)	59.9 ± 6.2	59.2 ± 6.8
**Sex (n (%))**		
Men	14 (61%)	15 (58%)
Women	9 (39%)	11 (42%)
Diabetes duration (years)	11.3 ± 6.4	10.9 ± 7.1
Alcohol use (no. (%))	9 (39%)	10 (39%)
**Clinical parameters**		
Body weight (kg)	98.4 ± 13.8	94.5 ± 13.1
BMI (kg/m^2^)	32.6 ± 4.4	31.6 ± 3.4
Total fat mass (%)	36.1 ± 10.3	36.6 ± 8.8
Hepatic triglyceride content (%)	18.1 ± 11.2	18.4 ± 9.4
**Fasting concentrations**		
CETP (µg/mL)	0.84 ± 0.22	0.81 ± 0.26
Total cholesterol (mmol/L)	4.82 ± 1.02	4.80 ± 1.02
HDL-cholesterol (mmol/L)	1.22 ± 0.25	1.30 ± 0.39
LDL-cholesterol (mmol/L)	2.60 ± 0.86	2.55 ± 0.91
Triglycerides (mmol/L)	2.19 ± 1.51	2.10 ± 1.09
Glucose (mmol/L)	8.7 ± 2.7	7.3 ± 1.7
HbA1c (%)	8.3 ± 1.1	8.1 ± 0.9
HbA1c (mmol/mol)	66.7 ± 11.5	64.7 ± 10.2
AST (IU/L)	31 ± 11	35 ± 21
ALT (IU/L)	15 ± 7	13 ± 5
**Concomitant drug use**		
Lipid lowering drugs (no. (%))	21 (91%)	19 (73%)
Metformin (g/day)	2.1 ± 0.7	2.0 ± 0.5
Sulfonylureas (no. (%))	6 (26%)	8 (31%)
Insulin (no. (%))	15 (65%)	17 (65%)
Insulin (units)	70 ± 46	69 ± 58

Results are presented as n (%) or mean ± SD. n = 49. Missing data: n = 2 for alcohol use and n = 2 for hepatic triglyceride content in placebo-group. ALT: alanine transaminase, AST: aspartate transaminase, CETP: cholesteryl ester transfer protein, HbA1c: glycated haemoglobin.

#### A decrease in hepatic triglyceride content was not accompanied by a change in circulating CETP

**Figure 1:**
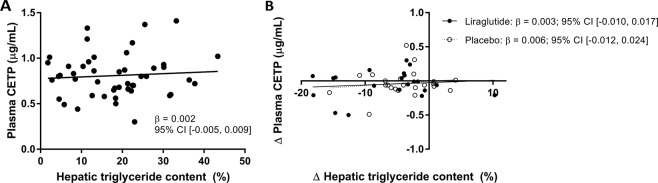
Associations between hepatic triglyceride content and plasma CETP levels in the MAGNA VICTORIA study. Hepatic triglyceride content in relation to plasma CETP level, n = 47 (**A**), and change of hepatic triglyceride content in relation to change in plasma CETP level, liraglutide n = 21, placebo n = 23 (**B**). CETP: cholesteryl ester transfer protein.

Treatment with liraglutide for 26 weeks decreased body weight in contrast to treatment with placebo (−4.3 ± 3.8 kg *vs* 0.1 ± 2.5 kg; mean change from baseline (liraglutide *vs* placebo): −4.5 kg; 95%CI [−6.4, −2.6]), as shown in Table 2. Furthermore, treatment with liraglutide decreased hepatic triglyceride content (−6.3%; 95%CI of difference [−9.5, −3.0]), but hepatic triglyceride content was also decreased in the placebo group (−4.0%; 95%CI [−6.0, −2.0]), without between-group differences (mean change from baseline (liraglutide *vs* placebo): −2.1%; 95%CI [−5.3, 1.0]). Interestingly, this decrease in hepatic triglycerides in both treatment groups was not accompanied by a decrease in circulating CETP after 26 weeks of intervention. Also, after 4 and 12 weeks of intervention, CETP was not affected in both the liraglutide group and the placebo group (not shown). Figure 1 shows that hepatic triglyceride content was not associated with CETP at baseline (β: 0.002 µg/mL per 1% increase in hepatic triglyceride content; 95%CI [−0.005, 0.009]). Furthermore, there was no association between the changes of both variables after treatment with liraglutide (β: 0.003 µg/mL; 95%CI [−0.010, 0.017]) or placebo (β: 0.006 µg/mL; 95%CI [−0.012, 0.024]). At baseline, circulating CETP concentration was lower in participants using lipid lowering drugs (0.80 ± 0.23 µg/mL; 82% of participants) than in participants not using lipid lowering drugs (0.93 ± 0.27 µg/mL; 18% of participants), mean difference 0.14 µg/mL; 95%CI of difference [−0.08, 0.35]). Furthermore, while HDL-cholesterol was not affected, total cholesterol, LDL-cholesterol and triglycerides were decreased in both groups. Finally, HbA1c was improved after treatment with liraglutide (−1.1%; 95%CI of difference [−1.5, −0.6]), but also after treatment with placebo (−0.7% 95%CI of difference [−1.1, −0.3]); without between-group differences (mean change from baseline (liraglutide *vs* placebo): -0.3%; 95%CI [−0.8, 0.2]).

**Table 2:** Body weight, hepatic triglyceride content and metabolic factors change from baseline to after 26 weeks of treatment in the MAGNA VICTORIA study.

Characteristic	Mean ± SD change from baseline to 26 weeks		Mean [95%CI] changes from baseline (liraglutide *vs* placebo)	P value
	Liraglutide (n = 22)	Placebo (n = 25)		
**Clinical parameters**				
Body weight (kg)	−4.3 ± 3.8	0.1 ± 2.5	−4.5 [−6.4, −2.6]	<0.001
BMI (kg/m^2^)	−1.5 ± 1.3	0.1 ± 0.8	−1.5 [−2.2, −0.9]	<0.001
Hepatic triglyceride content (%)	−6.3 ± 7.1	−4.0 ± 4.6	−2.1 (−5.3, 1.0)	0.174
**Metabolic factors**				
CETP (µg/mL)	−0.05 ± 0.20	−0.04 ± 0.18	0.00 [−0.10, 0.11]	0.977
Total cholesterol (mmol/L)	−0.72 ± 0.89	−0.46 ± 0.56	−0.22 [−0.59, 0.15]	0.231
HDL-cholesterol (mmol/L)	−0.02 ± 0.14	0.05 ± 0.25	−0.08 [−0.20, 0.05]	0.222
LDL-cholesterol (mmol/L)	−0.44 ± 0.51	−0.24 ± 0.52	−0.17 [−0.44, 0.10]	0.218
Triglycerides (mmol/L)	−0.50 ± 1.12	−0.60 ± 0.98	0.23 [−0.11, 0.56]	0.177
Glucose (mmol/L)	−1.7 ± 2.2	−0.6 ± 2.2	−0.5 [−1.7, 0.7]	0.434
HbA1c (%)	−1.1 ± 1.0	−0.7 ± 0.9	−0.3 [−0.8, 0.2]	0.265
HbA1c (mmol/mol)	−11.6 ± 11.1	−7.7 ± 9.4	−2.9 [−8.1, 2.3]	0.265
AST (IU/L)	−6 ± 11	−12 ± 22	2 [−3, 6]	0.459
ALT (IU/L)	16 ± 12	14 ± 10	1 [−5, 7]	0.778

Results are presented as mean ± SD. n = 47. Missing data: n = 2 for hepatic triglyceride content in the placebo group, n = 1 for hepatic triglyceride content, n = 1 for LDL-cholesterol and n = 1 for glucose in the liraglutide group. ALT: alanine transaminase, AST: aspartate transaminase, CETP: cholesteryl ester transfer protein, HbA1c: glycated haemoglobin.

### Population-based study

#### Population characteristics

Demographic and clinical characteristics of the NEO study population are presented in Table 3. While mean BMI was lower in women than in men, mean total body fat was higher in women. Hepatic triglyceride content ranged from 0.2% to 62.9% and was lower in women than in men. Serum CETP ranged from 0.88 to 5.02 µg/mL. Men had a lower serum CETP concentration (2.38 ± 0.62 µg/mL) than women (2.64 ± 0.64 µg/mL) (difference: −0.26; 95%CI [−0.17, −0.35]), and CETP concentration was lower in participants using lipid lowering drugs (2.13 ± 0.72 µg/mL) compared with participants not using lipid lowering drugs (2.56 ± 0.62 µg/mL) (difference −0.43; 95%CI [−0.54, −0.32]).

**Table 3:** Characteristics of participants from the NEO study population who underwent proton magnetic resonance spectroscopy of the liver to assess hepatic triglyceride content, stratified by sex.

Characteristic	Men	Women
Proportion of participants (%)	45	55
Ethnicity (% whites)	95	95
Age (year)	55.8 ± 6.5	55.1 ± 5.5
Alcohol use (% users)	89	82
Alcohol intake (g/day)	12.1 ± 9.5	5.6 ± 5.0
BMI (kg/m^2^)	26.4 ± 3.5	25.4 ± 4.2
Total body fat (%)	24.2 ± 5.7	36.3 ± 6.1
***Fasting concentrations***		
CETP (µg/mL)	2.38 ± 0.62	2.64 ± 0.64
Total cholesterol (mmol/L)	5.56 ± 1.00	5.81 ± 1.03
HDL-cholesterol (mmol/L)	1.31 ± 0.36	1.72 ± 0.43
LDL-cholesterol (mmol/L)	3.62 ± 0.93	3.59 ± 0.95
Triglycerides (mmol/L)	1.38 ± 0.89	1.09 ± 0.68
ALT (IU/L)	28.4 ± 13.0	21.3 ± 7.7
AST (IU/L)	25.8 ± 7.3	22.9 ± 6.2
HbA1c (%)	5.4 ± 0.6	5.3 ± 0.3
HbA1c (mmol/mol)	35.5 ± 6.7	34.7 ± 3.6
***Comorbidity and medication***		
Hepatic triglyceride content (%)	3.4 (1.9, 7.7)	1.7 (1.1, 4.5)
Diabetes (% yes)	6	4
Impaired fasting glucose (% yes)	10	5
Oral glucose lowering drugs (% users)	3	1
Insulin (% users)	0.04	0.5
Oral glucose lowering drugs and insulin (% users)	0.8	0.1
Cardiovascular disease (% yes)	5	4
Lipid lowering drugs (% users)	12	5

Results were based on analyses weighted towards the BMI distribution of the general population (n = 1,611) and presented as mean ± SD, median (interquartile range) or percentage. Missing data: n = 2 for ethnicity, n = 1 for total body fat, n = 5 for plasma total cholesterol, HDL-cholesterol, LDL-cholesterol, triglycerides, ALT and AST concentrations, n = 15 for HbA1c concentration, n = 6 for presence of diabetes, n = 5 for presence of cardiovascular disease. ALT: alanine transaminase, AST: aspartate transaminase, BMI: body mass index, CETP: cholesteryl ester transfer protein, HbA1c: glycated haemoglobin, HDL: high-density lipoprotein, IU: international unit, LDL: low-density lipoprotein, NEO: Netherlands Epidemiology of Obesity.

#### Hepatic triglyceride content was not associated with circulating CETP in the general population

**Figure 2:**
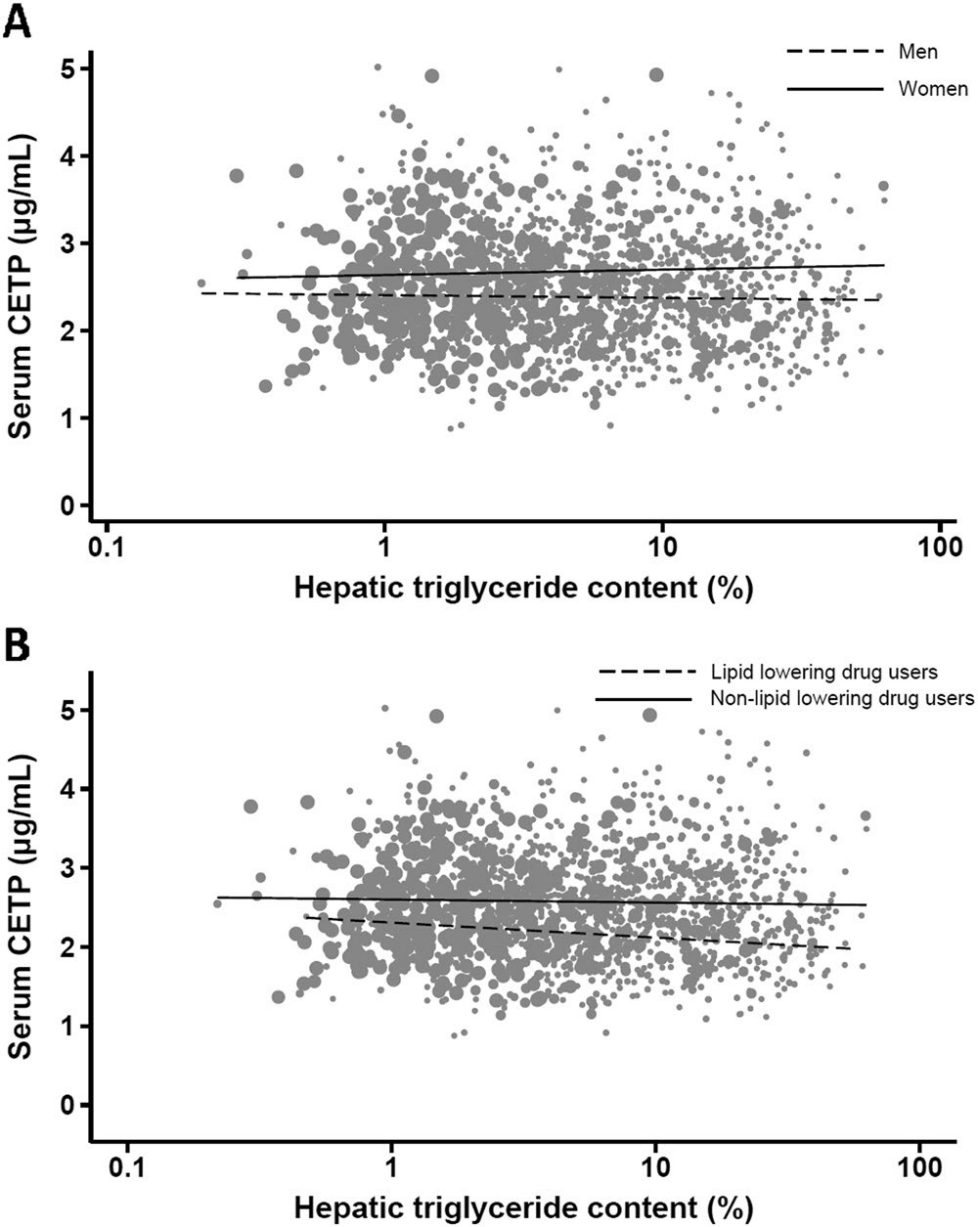
Associations between hepatic triglyceride content and serum CETP concentrations in the NEO study population. Crude associations between hepatic triglyceride content and serum CETP concentration, stratified by sex (**A**) and lipid lowering drug use (**B**). Results were based on analyses weighted towards the BMI distribution of the general population (n = 1,611). CETP: cholesteryl ester transfer protein; NEO: Netherlands Epidemiology of Obesity.

As shown in Fig. 2, hepatic triglyceride content was not associated with serum CETP concentration in the NEO study, neither in men, women, lipid lowering drug users or non-users of lipid lowering drugs. As can be appreciated from Suppl. Table 1, a 10% increase in hepatic triglyceride content was associated with a −0.001 µg/mL (95%CI [−0.005, 0.003]) difference in serum CETP. Similar associations around the null were observed after adjustment for confounding variables.

## Discussion

In this double-blind, randomised placebo-controlled trial, we observed that a reduction in hepatic triglyceride content after treatment with liraglutide, but also with placebo, was not accompanied by a reduction in circulating CETP concentration. In line, we found no evidence for an association between hepatic triglyceride content and circulating CETP concentration in a large population-based cohort study (n = 1,611). Our findings imply that plasma CETP concentration is not determined by hepatic triglyceride content.

Our observation that the hepatic triglyceride content decreased after liraglutide treatment is in accordance with results from previous studies in humans and rodents^8,11^. However, this decrease was also observed in the placebo group. Furthermore, although the GLP-1 analogue exendin-4 decreased hepatic *CETP* gene expression and CETP concentration in rodents^12^, we found no effect of liraglutide treatment on the circulating CETP concentration. These data are corroborated by our finding that hepatic triglyceride content was not associated with circulating CETP concentration in the large population-based NEO cohort. There was also no association present after stratification by lipid lowering drug use, which implies that lipid lowering drugs do not mediate or influence the effects of hepatic triglyceride content on CETP concentration. Nevertheless, our data seem counterintuitive, as previous studies did report a decrease in CETP upon other interventions that reduce hepatic steatosis. For example, Jonker *et al*.^18^ showed that the PPARγ agonist pioglitazone decreased hepatic triglyceride content (from 6 to 4%) as well as CETP concentration (-12%), while the decrease of circulating CETP correlated with the decrease in hepatic triglyceride content. Notably, in their study both the hepatic triglyceride and circulating CETP concentration at baseline were lower compared to our study and the reduction of hepatic triglyceride content induced by pioglitazone was smaller than the decrease in the liraglutide group in the present study. In another study, patients with type 2 diabetes received a 16-week very low calorie diet, which resulted in dramatic weight loss and a large reduction in hepatic triglyceride content (from 21 to 3%) as well as CETP concentration (−18%)^7^. After gastric banding surgery, with associated substantial weight loss, similar large reductions in CETP concentration have been described^3,19^.

Interestingly, hepatic triglyceride content was not only decreased in the liraglutide-treated group, but also in the placebo group. This is probably due to treatment of participants in both groups according to current clinical guidelines, which resulted in an intensified treatment with glucose-lowering drugs in the placebo-group, while in the liraglutide-group such treatment could be decreased. Both groups showed improved glucoregulation, evidenced by decreased HbA1c after treatment. It is thus likely that intensified treatment of the placebo group decreased the rate of hepatic lipogenesis and consequently lowered intrahepatic triglyceride storage, resulting in increased hepatic insulin sensitivity that lowers glucose production^20,21^. Notably, similar to treatment with liraglutide, the reduction in hepatic triglycerides caused by placebo treatment was not accompanied by a reduction in circulating CETP.

It is interesting to speculate on the mechanism by which GLP-1 analogues may influence plasma CETP concentration in humans. Notably, in contrast to previous studies^7,18^, our findings show that liraglutide and placebo-induced lowering of hepatic triglycerides is not accompanied by decreases in plasma CETP concentration. We have previously shown that the GLP-1 analogue exendin-4 decreased plasma CETP concentration, which was accompanied by a reduction in the number of hepatic macrophages^12^, the main source of CETP production. The reduction in hepatic macrophage content by exendin-4 attributes to reduced macrophage recruitment from the circulation and/or enhanced macrophage elimination from the liver. Indeed, in rodents, exendin-4 decreased the hepatic gene expression of monocyte chemotactic protein-1^11,12^, which mediates monocyte/macrophage recruitment from the circulation to tissue. Since Panjwani *et al*.^22^ reported that the GLP-1 receptor is undetectable in isolated macrophages and hepatocytes, the effect of exendin-4 on hepatic macrophage content is unlikely mediated via the GLP-1 receptor. However, the exact action of exendin-4 on macrophage recruitment and elimination is unclear, and whether other GLP-1 analogues exert the same beneficial effects on hepatic macrophage content needs to be further investigated. It is possible that this is an effect specific for exendin-4 not shared by liraglutide. Furthermore, it is possible that, in contrast to in rodents, GLP-1 receptor agonists in general, or the applied dose of liraglutide specifically, fail to reduce the hepatic macrophage content in humans. Indeed, in the LEAN-trial, in which patients with NASH were treated with liraglutide, no effects were observed on lobular inflammation and overall non-alcoholic fatty liver disease (NAFLD) activity score^8^. Collectively, it is likely that liraglutide fails to affect hepatic macrophages to an extent that is sufficient to decrease CETP concentration.

In this light, it is interesting to note that we previously studied the association of metabolic liver inflammation with hepatic and circulating CETP^23^. We showed that metabolic liver inflammation, as a histologically determined component of NAFLD in obese individuals, did not associate with CETP measures (i.e. liver *CETP*, liver CETP positive cells and circulating CETP concentrations). These data are in line with the findings of the current study, as apparently, metabolic triggers of liver damage do not decrease CETP production. Interestingly, infection-related liver inflammation, as induced by Gram-negative bacteria, strongly decreases CETP production by the liver^24^. As both metabolic induced liver steatosis and inflammation^23^ do not affect CETP production by Kupffer cells, it seems that NAFLD does not mimic the strong effects of Gram-negative bacterial infections on the hepatic expression and production of CETP by Kupffer cells. This suggests that metabolically-induced and infection-related inflammation may have different effects on the expression and production of CETP by Kupffer cells.

Preclinical studies using mice and cultured cells have shown that the Liver X Receptor α (LXRα) plays a crucial role in regulation CETP expression. The natural ligands of LXRα are oxysterols^25,26^. In line, it has been shown that lipid lowering drugs decrease total hepatic cholesterol content and levels of oxysterols, thereby diminishing *LXRα* activation and CETP expression in CETP-transgenic mice^27^. Therefore, the lower levels of circulating CETP in participants using lipid lowering drugs in our intervention trial as well as in our cohort study are likely explained by attenuated CETP expression by reduced oxysterol-mediated LXRα signalling. It has previously been proposed that a decreased hepatic triglyceride content upon an intervention with caloric restriction or pioglitazone may reduce CETP concentration via this LXRα-dependent mechanism, as a reduction in triglycerides would be accompanied by a reduction in the natural LXRα agonists^7,18^. However, since in our trial a reduction of hepatic triglyceride content was not accompanied by a reduction of CETP concentration, our results imply that interventions on hepatic triglyceride content *per se* do not affect hepatic oxysterols and thereby *LXRα*-mediated CETP production. With these new insights, we speculate that decreased CETP concentration previously found after interventions with pioglitazone and caloric restriction might be better explained by a reduction of the number of Kupffer cells than by a decreased hepatic oxysterol content.

The strengths of this study are the randomised placebo-controlled trial design, and the availability of data on hepatic triglyceride content and CETP concentration from a large population-based cohort. A limitation is that the study population of the NEO study was predominantly white and results may therefore not apply to other ethnical groups. Furthermore, the study design of the trial, in which in addition to study medication patients received treatment according to current clinical guidelines, is another possible limitation, since we were not able to investigate the effects of liraglutide only. Also, use of co-medication could interfere with the effects of liraglutide on CETP. Nevertheless, this study design increases the generalizability of our findings.

In summary, in a randomised placebo-controlled trial, we showed that liraglutide treatment and placebo intervention on top of standard treatment with glucose lowering drugs decrease hepatic triglyceride content without decreasing plasma CETP concentration. We confirmed the absence of an association between hepatic triglyceride content and CETP in a large population-based cohort study. This implies that circulating CETP concentration is not determined by hepatic triglyceride content.

## Supplementary information

Supplementary information

## Data Availability

The datasets generated and/or analysed during the current study are not publicly available but are available from the corresponding author on reasonable request.
