# Supplementary material for: Hepatic triglyceride content does not affect circulating CETP: lessons from a liraglutide intervention trial and a population-based cohort
Source: Sci Rep. 2019 Jul 10;9:9996. doi: 10.1038/s41598-019-45593-2 (PMC6620358; doi:10.1038/s41598-019-45593-2)
Supplement: Supplementary file 1 — Supplementary information [file 41598_2019_45593_MOESM1_ESM.docx]

**Supplementary information**

**Hepatic triglyceride content does not affect circulating CETP: lessons from a liraglutide intervention trial and a population-based cohort**

***Short title: Hepatic TG content does not affect CETP***

Huub J. van Eyk (MSc)^a,b,#,*^, Lisanne L. Blauw (PhD)^a,c,#^, Maurice B. Bizino (MSc)^a,d^, Yanan Wang (PhD)^a,b^, Ko Willems van Dijk (PhD)^a,b,e^, Renée de Mutsert (PhD)^c^, Johannes W.A. Smit (PhD)^f^, Hildo J. Lamb (PhD)^d^, Ingrid M. Jazet (PhD)^a,b^, Patrick C.N. Rensen (PhD)^a,b^

^a^Dept. Medicine, Div. Endocrinology, Leiden University Medical Center (LUMC), Leiden, the Netherlands, ^b^Einthoven Laboratory for Experimental Vascular Medicine, LUMC, Leiden, the Netherlands, ^c^Dept. Epidemiology, LUMC, Leiden, the Netherlands, ^d^Dept. Radiology, LUMC, Leiden, the Netherlands, ^e^Dept. Human Genetics, LUMC, Leiden, the Netherlands, ^f^Dept. Medicine, Radboud University Medical Center, Nijmegen, the Netherlands.

^#^Shared first author

**Supplementary Materials and Methods Netherlands Epidemiology of Obesity (NEO) study**

*Covariates*

Questionnaires were sent to all participants and completed at home. The general questionnaire included questions on demographic, lifestyle and clinical information. Smoking status was categorized into never smoker, former smoker and current smoker. Menopausal status was classified as premenopausal, perimenopausal (menopausal during last year) or postmenopausal. Participants were classified as having pre-existing cardiovascular disease when a medical history of myocardial infarction, angina, congestive heart failure, stroke or peripheral vascular disease was reported via the questionnaire. Participant were classified as having diabetes when the disease was self-reported via the questionnaire or when anti-diabetic medication was used. Research nurses recorded current medication use. Alcohol intake was assessed with a semi-quantitative food frequency questionnaire (FFQ)^1^, and calculated from the FFQ using the 2011 version of the Dutch food composition table (NEVO-2011).

Body weight and total body fat were determined with the Tanita bio-impedance balance (TBF-310, Tanita International Division, UK) without shoes and with subtraction of one kilogram (kg) to correct for the weight of clothing. BMI was calculated by dividing the weight in kilograms by the height in meters squared.

Fasting serum total cholesterol and TG concentrations were measured with enzymatic colorimetric assays (Roche Modular P800 Analyzer, Roche Diagnostics, Mannheim, Germany) and fasting serum HDL-cholesterol concentrations with third generation homogenous HDL-cholesterol methods (Roche Modular P800 Analyzer, Roche Diagnostics, Mannheim, Germany). Fasting LDL-cholesterol concentrations were calculated using the Friedewald equation^2^. HbA1c was assessed with Boronate affinity high-performance liquid chromatography (HPLC) (Primus Ultra, Siemens Healthcare Diagnostics, Breda, the Netherlands).

*Statistical analyses: weighting*

In the NEO study, individuals with a BMI of 27 kg/m^2^ or higher were oversampled. Men and women aged between 45 and 65 years with a self-reported BMI of 27 kg/m^2^ or higher were eligible to participate. From one nearby municipality (Leiderdorp, the Netherlands) all inhabitants aged between 45 and 65 years were invited to participate regardless of their BMI, in order to obtain a reference distribution for BMI. To correctly represent baseline associations in the general population^3^, adjustments for the oversampling of individuals with a BMI ≥27 kg/m^2^ were made in the analyses. This was done by weighting all participants towards the BMI distribution of participants from the Leiderdorp municipality^4^, whose BMI distribution was similar to the BMI distribution of the general Dutch population in the age range of 45-65 years^5^. In practice, this means that participants with a lower BMI were assigned larger weight factors than participants with a higher BMI in the analyses. All results are based on weighted analyses, and therefore apply to a population-based study without oversampling of individuals with overweight or obesity. As a consequence, the weighted characteristics of the population are expressed in proportions instead of absolute numbers.

**Supplementary table 1.** NEO study: Difference in serum CETP concentration per 10% relative increase in hepatic triglyceride content.

| **Model** | **Difference in serum CETP concentration (µg/mL)^a^** | **95%CI** |
| --- | --- | --- |
| 1 | -0.001 | -0.005, 0.003 |
| 2 | 0.003 | -0.002, 0.007 |
| 3 | 0.003 | -0.001, 0.007 |

Results were based on analyses weighted towards the BMI distribution of the general population (n=1,611). Model 1: unadjusted. Model 2: adjusted for age, sex. Model 3: adjusted for age, sex, ethnicity, smoking status, alcohol intake and physical activity. Missing data: n=2 for ethnicity, n=37 for physical activity. ^a^ (Beta coefficients from linear regression)*ln(1.1); difference per 10% relative increase in hepatic triglyceride content. BMI: body mass index, CETP: cholesteryl ester transfer protein, NEO: Netherlands Epidemiology of Obesity.

**References**

**1.** Feunekes GI, Van Staveren WA, De Vries JH, Burema J, Hautvast JG. Relative and biomarker-based validity of a food-frequency questionnaire estimating intake of fats and cholesterol. *The American journal of clinical nutrition.* 1993;58:489-496.

**2.** Friedewald WT, Levy RI, Fredrickson DS. Estimation of the concentration of low-density lipoprotein cholesterol in plasma, without use of the preparative ultracentrifuge. *Clinical chemistry.* 1972;18:499-502.

**3.** Korn EL, Graubard BI. Epidemiologic studies utilizing surveys: accounting for the sampling design. *American journal of public health.* 1991;81:1166-1173.

**4.** Lumley T. Analysis of complex survey samples. <http://www.jstatsoft.org/v09/i08/paper>*.* Vol 20132004 [accessed 02.05.13].

**5.** Llewellyn-Smith IJ. Anatomy of synaptic circuits controlling the activity of sympathetic preganglionic neurons. *Journal of chemical neuroanatomy.* 2009;38:231-239.
